# Midostaurin Reverses ABCB1-Mediated Multidrug Resistance, an *in vitro* Study

**DOI:** 10.3389/fonc.2019.00514

**Published:** 2019-06-18

**Authors:** Ning Ji, Yuqi Yang, Chao-Yun Cai, Jing-Quan Wang, Zi-Ning Lei, Zhuo-Xun Wu, Qingbin Cui, Dong-Hua Yang, Zhe-Sheng Chen, Dexin Kong

**Affiliations:** ^1^Tianjin Key Laboratory on Technologies Enabling Development of Clinical Therapeutics and Diagnostics, School of Pharmacy, Tianjin Medical University, Tianjin, China; ^2^Department of Pharmaceutical Sciences, College of Pharmacy and Health Sciences, St. John's University, Queens, NY, United States; ^3^Research Center, School of Medicine, Tianjin Tianshi College, Tianyuan University, Tianjin, China

**Keywords:** midostaurin, multidrug resistance, ATP-binding cassette (ABC) transporter, ABC, chemotherapy

## Abstract

Overexpression of ABC transporters in cancer cells is an underlying mechanism of multidrug resistance (MDR), leading to insensitive response to chemotherapeutic strategies. Thus, MDR is often results in treatment failure in the clinic. In this study, we found midostaurin, a Food and Drug Administration (FDA)-approved anti-leukemia drug, can antagonize ATP-binding cassette subfamily B member 1 (ABCB1)-mediated MDR. Our results indicated that midostaurin has the capacity to antagonize ABCB1-mediated MDR, while no significant reversal effect was found on ATP-binding cassette subfamily G member 2 (ABCG2)-mediated MDR. Our subsequent resistance mechanism studies showed that midostaurin directly inhibited the efflux function of the ABCB1 transporter without alteration of the expression level or the subcellular localization of ABCB1 transporter. In addition, midostaurin inhibited the ATPase activity of ABCB1 transporter in a dose-dependent manner. Moreover, our *in silico* docking study predicted that midostaurin could interact with the substrate-binding sites of ABCB1 transporter. This novel finding could provide a promising treatment strategy that co-administrating midostaurin with anticancer drugs in the clinic could overcome MDR and improve the efficiency of cancer treatment.

## Introduction

Multidrug resistance (MDR) in cancer, a phenomenon leading to synchronous resistance of cancer cells to structurally unrelated antineoplastic drugs, is one of the most critical factors responsible for the failure of chemotherapeutics and the poor survival rate of patients (1). Several mechanisms are involved in cancer MDR, including reduced apoptosis, advanced DNA damage repair mechanisms, or altered drug metabolism. However, the most prominent factor is ABC transporter-mediated efflux of antineoplastic drugs (3, 51).

The transport system superfamily of ABC transporters plays critical roles in physiological and pharmacological processes (2). The human ABC protein family has been divided into seven subfamilies (ABCA to ABCG). The ABC transporter family has 49 ABC proteins and 48 of them have identified functions (3, 4). As one of the main contributors, ABCB1 (P-gp/MDR1) is widely expressed not only in the placenta, but in the blood-brain barrier (BBB), intestines, livers and kidneys, in order to protect the body from xenobiotics (5, 6). The ABCB1 transporter also mediates the transport of a wide range of physiological substrates like lipids, porphyrins, and sterols (7). Furthermore, a broad range of chemotherapeutic drugs are substrates of the ABCB1 transporter, such as taxanes and anthracyclines. ABCB1 transporter significantly increases the efflux of such anticancer drugs, a major reason leading to ABCB1-mediated MDR (8). It has been documented that ABCB1 is strongly related to the chemotherapy prognosis and the progression of malignancy (9). Thus, it is critical to elude MDR by either decreasing the expression level of ABCB1 proteins or inhibiting the efflux function of ABCB1 through specific and potent inhibitors.

Midostaurin, a multi-kinase inhibitor that was originally developed as a protein kinase C (PKC) inhibitor for treatment of patients with solid malignancy (10), has already been approved by the FDA for treatment of acute myelocytic leukemia (AML) with Fms-like tyrosine kinase 3 (FLT3)-mutant subtype (11). It has recently been reported that the combination of midostaurin with standard chemotherapy can significantly prolong overall and event-free survival in patients who suffer from AML with a FLT3 mutation (12). Here, we report the reversal effects of midostaurin on ABCB1-mediated MDR when co-administrated with conventional antineoplastic drugs.

## Materials and Methods

### Chemicals

Midostaurin was obtained from Thermo Fisher Scientific Inc. (Rockford, IL). Bovine serum albumin (BSA), fetal bovine serum (FBS), Dulbecco's modified Eagle's Medium (DMEM), penicillin/streptomycin and 0.25% trypsin were products from Corning Incorporated (Corning, NY). The monoclonal antibody for GAPDH (catalog number MA5-15738, lot number SA247966, clone GA1R), Alexa Fluor 488 conjugated goat anti-mouse IgG secondary antibody, were purchased from Thermo Fisher Scientific Inc. (Rockford, IL). Paclitaxel, doxorubicin, colchicine, cisplatin, mitoxantrone, verapamil, the monoclonal antibodies for ABCB1 (catalog number P7965, lot number 067M4761V, clone F4), dimethylsulfoxide (DMSO), 3-(4,5-dimethylthiazol-yl)-2,5-diphenyltetrazolium bromide (MTT), Triton X-100, 4',6-diamidino-2-phenylindole (DAPI), and paraformaldehyde, were obtained from Sigma-Aldrich (St. Louis, MO). HRP-conjugated rabbit anti-mouse IgG secondary antibody (catalog number 7076S, Lot number 32) were obtained from Cell Signaling Technology Inc. (Danvers, MA). Ko143 was a product from Enzo Life Sciences (Farmingdale, NY). [^3^H]-paclitaxel (15 Ci/mmol) was purchased from Moravek Biochemicals, Inc. (Brea, CA). All other chemicals were purchased from Sigma Chemical Co (St. Louis, MO).

### Cell Lines and Cell Culture

The ABCB1-overexpressing KB-C2 cell line was created by gradually adding colchicine to parental human epidermoid carcinoma KB-3-1 cells, and was kindly provided by Dr. Shin-ichi Akiyama (Kagoshima University, Kagoshima, Japan). The KB-C2 line was cultured in medium containing 2 μg/mL colchicine (13) to maintain its drugresistant characteristics. The SW620/Ad300 cells were cultured in medium with 300 ng/mL doxorubicin (14). KB-3-1, KB-C2, SW620, and SW620/Ad300 cells were used for ABCB1 reversal study. The human non-small cell lung cancer (NSCLC) NCI-H460 cell line and its subline of ABCG2-overexpressing NCI-H460/MX20 cells were used for ABCG2 reversal study. The NCI-H460/MX20 cells were selected by using a high dose of mitoxantrone and maintained in medium with 20 ng/mL mitoxantrone (15). HEK293/pcDNA3.1 and HEK293/ABCB1 were established by transfecting the human embryonic kidney HEK293 cells with empty and ABCB1 expressing vector, respectively (16). SW620 and SW620/Ad300 cells, NCI-H460 and NCI-H460/MX20 cells, were kindly provided by Drs. Susan Bates and Robert Robey (NCI, NIH, Bethesda, MD). HEK293/ABCB1 were kindly provided by Dr. Suresh V. Ambudkar (NCI, NIH, Bethesda, MD). All aforementioned cell lines were maintained in DMEM medium containing 10% fetal bovine serum and 1% penicillin/streptomycin at 37°C in a humidified atmosphere containing 5% CO_2_. All cells were grown as an adherent monolayer and drug-resistant cells were grown in drug-free culture media for more than 20 days before assay.

### MTT Cytotoxicity Assay

Cell viability was determined by MTT assay as we previously described (17). Each type of cell was harvested and resuspended before being seeded onto a 96-well plate at a final quantity of 5 ×10^3^ cells per well in 160 μL of medium, and was then incubated overnight. Midostaurin and positive control drugs were added 2 h prior to incubation with or without anticancer drugs. After 72 h of further incubation, MTT solution (4 mg/mL) was added to each well and the cells were incubated for an additional 4 h at 37°C. Subsequently, the supernatant was discarded and 100 μL of DMSO was added to each well in order to dissolve the formazan crystals. An accuSkanTM GO UV/Vis Microplate Spectrophotometer from Fisher Sci. (Fair Lawn, NJ) was used to determine the absorbance at 570 nm. The concentration for 50% inhibition of cell viability (IC_50_) of the anticancer drug was calculated as previously described (18). For positive control drugs, verapamil (3 μM) and Ko143 (3 μM) were used as reference inhibitors to reverse ABCB1- and ABCG2-mediated MDR, respectively. Cisplatin, which is not a substrate of ABCB1 or ABCG2, was used as a negative control chemotherapeutic drug.

### Western Blotting and Immunofluorescence Analysis

Western blotting analysis was performed as previously described (19). Briefly, cells were lysed after incubated with or without midostaurin (500 nM) for varying amounts of time (0, 24, 48, and 72 h). The concentration of protein was determined by BCA Protein Assay Kit from Pierce (Rockford, IL). Equal amounts (20 μg) of proteins were subjected to 10% sodium dodecyl sulfate polyacrylamide gel electrophoresis (SDS-PAGE) and transferred to PVDF membranes from Millipore (Billerica, MA). The presence of ABCB1 was determined using monoclonal antibody F4 (dilution 1:500). GAPDH was used as a loading control. The resulting protein bands were analyzed using Image J software. The immunofluorescence assay was performed as previously described (17). Briefly, after being cultured overnight in 24-well plates, cells (2 ×10^4^/well) were treated with midostaurin for 72 h at 500 nM concentration. Then, cells were fixed in 4% paraformaldehyde for 10 min and permeabilized by 0.1% Triton X-100 for 10 min before being blocked with 6% BSA for 1 h at 37°C. The presence of ABCB1 was determined using monoclonal antibody F4 (dilution 1:100) for incubation at 4°C overnight. Cells were washed with iced PBS after each incubation time. Alexa Fluor 488 (Ex = 499 nm, Em = 519 nm) conjugated secondary antibody (1:1,000) was used after washing with iced PBS. DAPI (Ex = 345 nm, Em = 455 nm) was used to counterstain the nuclei. The cells were washed with ice-cold PBS before being imaged. Immunofluorescence images were collected using an EVOS FL Auto fluorescence microscope from Life Technologies Corporation (Gaithersburg, MD).

### Doxorubicin Accumulation and Fluorescence Microscopic Analysis

Cells were grown in 6-well plates and washed twice with phosphate-buffered saline (PBS) before the pre-treatment of 500 nM of midostaurin. After 1 h of midostaurin pretreatment, 10 μM of doxorubicin was then added to each well for further incubation (1 h). Immunofluorescence images were collected using an EVOS FL Auto fluorescence microscope from Life Technologies Corporation (Gaithersburg, MD). Excitation and emission wavelengths of doxorubicin were 475 and 585 nm, respectively.

### [^3^H]-Paclitaxel Accumulation and Efflux Assay

We conducted [^3^H]-paclitaxel accumulation assay using KB-3-1 and its drug-resistant subline KB-C2 cells. As previously described (20), 5 ×10^5^ cells/well were cultured in 24-well plates overnight before the assay, and midostaurin was added 2 h prior to the addition of [^3^H]-paclitaxel. After incubating with [^3^H]-paclitaxel with or without midostaurin for 2 h at 37°C, cells were washed twice with iced PBS, and lysed with 0.25% trypsin before being placed in 5 mL scintillation fluid, and radioactivity was measured in the Packard TRI-CARB 1900CA liquid scintillation analyzer from Packard Instrument (Downers Grove, IL).For the efflux assay, KB-3-1 and KB-C2 cells (20) were incubated with midostaurin for 2 h followed by incubation with [^3^H]-paclitaxel, with or without midostaurin for 2 h at 37°C. The cells were washed with iced PBS twice and then lysed at various time points (0, 30, 60, and 120 min) with trypsin. Subsequently, cells were placed in 5 mL of scintillation fluid and radioactivity was measured in the Packard TRI-CARB 1900CA liquid scintillation analyzer from Packard Instrument (Downers Grove, IL).

### ATPase Assay

The ABCB1-associated ATPase activities were measured using PREDEASY ATPase Kits from TEBU-BIO nv (Boechout, Belgium) with modified protocols. Briefly, cell membranes that overexpressed ABCB1 were thawed and diluted before use. Sodium orthovanadate (Na_3_VO_4_) was used as an ATPase inhibitor. Various concentrations of midostaurin were incubated with membranes for 5 min. The ATPase reactions were initiated by adding 5 mM Mg^2+^-ATP. Luminescence signals of P_i_ were initiated and measured after incubation at 37°C for 40 min with brief mixing. The changes of relative light units were determined by comparing Na_3_VO_4_-treated samples with midostaurin-treated groups.

### Molecular Modeling of Human ABCB1 Homology Model

In silico docking analysis was conducted using software Maestro 11.5 (Schr'´odinger, LLC, New York, NY, 2018) (21). Human ABCB1 homology model was established by Dr. Aller based on refined mouse ABCB1 (PDB ID: 4M1M) (22). Afterwards, the docking grid at the drug-binding pocket was generated (23). The ligand was essentially prepared to perform glide XP docking with the default protocols.

### Statistical Analysis

All data are expressed as the mean ± SD and were analyzed using one-way ANOVA. All experiments were repeated at least three times. Differences were considered significant when *P* < 0.05.

## Results

### Midostaurin Significantly Antagonized ABCB1-Mediated MDR in ABCB1-Overexpressing Cancer Cells

**Figure 1:**
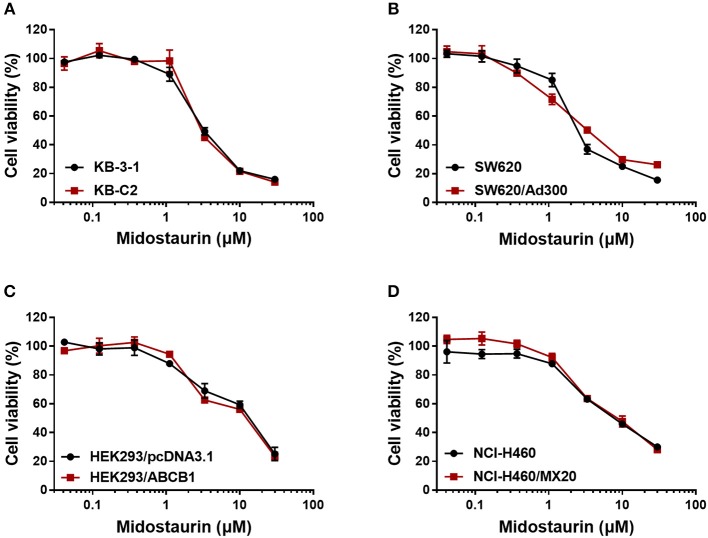
Dose-viability curves of cells used in this study incubated with midostaurin for 72 h. Dose-viability curves for **(A)** KB-3-1 and KB-C2, **(B)** SW620 and SW620/Ad300, **(C)** HEK293/pcDNA3.1 and HEK293/ABCB1, and **(D)** NCI-H460 and NCI-H460/MX20.

Firstly, to avoid cytostatic-induced reversal phenomenon, we conducted MTT assays to evaluate the cytostatic effects of midostaurin in the ABCB1-overexpressing cells and corresponding parental cells that we would use. Hence, we could choose concentrations that would not significantly influence cell viability. We conducted further experiments with 200 and 500 nM doses of midostaurin (Figure 1).

**Figure 2:**
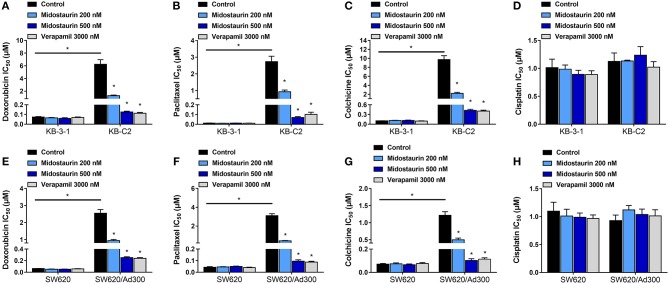
The reversal effect of midostaurin on ABCB1-mediated MDR in ABCB1-overexpression cancer cells. IC_50_ values of **(A)** doxorubicin, **(B)** paclitaxel, **(C)** colchicine, and **(D)** cisplatin in parental KB-3-1 and drug-selected ABCB1-overexpression resistant KB-C2 cells with or without treatment of midostaurin. IC_50_ of **(E)** doxorubicin, **(F)** paclitaxel, **(G)** colchicine, and **(H)** cisplatin in parental SW620 and drug-selected ABCB1-overexpression resistant SW620/Ad300 cells with or without treatment of midostaurin. Data are expressed as mean ± SD, representative of at least three independent experiments. ^*^P < 0.05, compared with control group.

As shown in Figure 2, midostaurin significantly sensitized ABCB1-overexpressing cancer cells KB-C2 (Figures 2A–C) and SW620/Ad300 (Figures 2E–G) to ABCB1 substrates (doxorubicin, paclitaxel, and colchicine), compared with their control resistance cells, and this sensitization occurred in a dose-dependent manner. At 200 or 500 nM, midostaurin could not alter the IC_50_ values of the above chemotherapeutic drugs to parental KB-3-1 (Figures 2A–C) and SW620 (Figures 2E–G) cells. In addition, when combined with cisplatin, a platinum drug which is known to not be a substrate of ABCB1, midostaurin showed no significant difference in its cytotoxic effect in neither the resistant cell lines nor the parental cell lines (Figures 2D,H). In this study, verapamil, a potent ABCB1 inhibitor, was used as a positive control drug (24).

### Midostaurin Significantly Antagonized ABCB1-Mediated MDR in ABCB1-Gene-Transfected Cells

**Figure 3:**
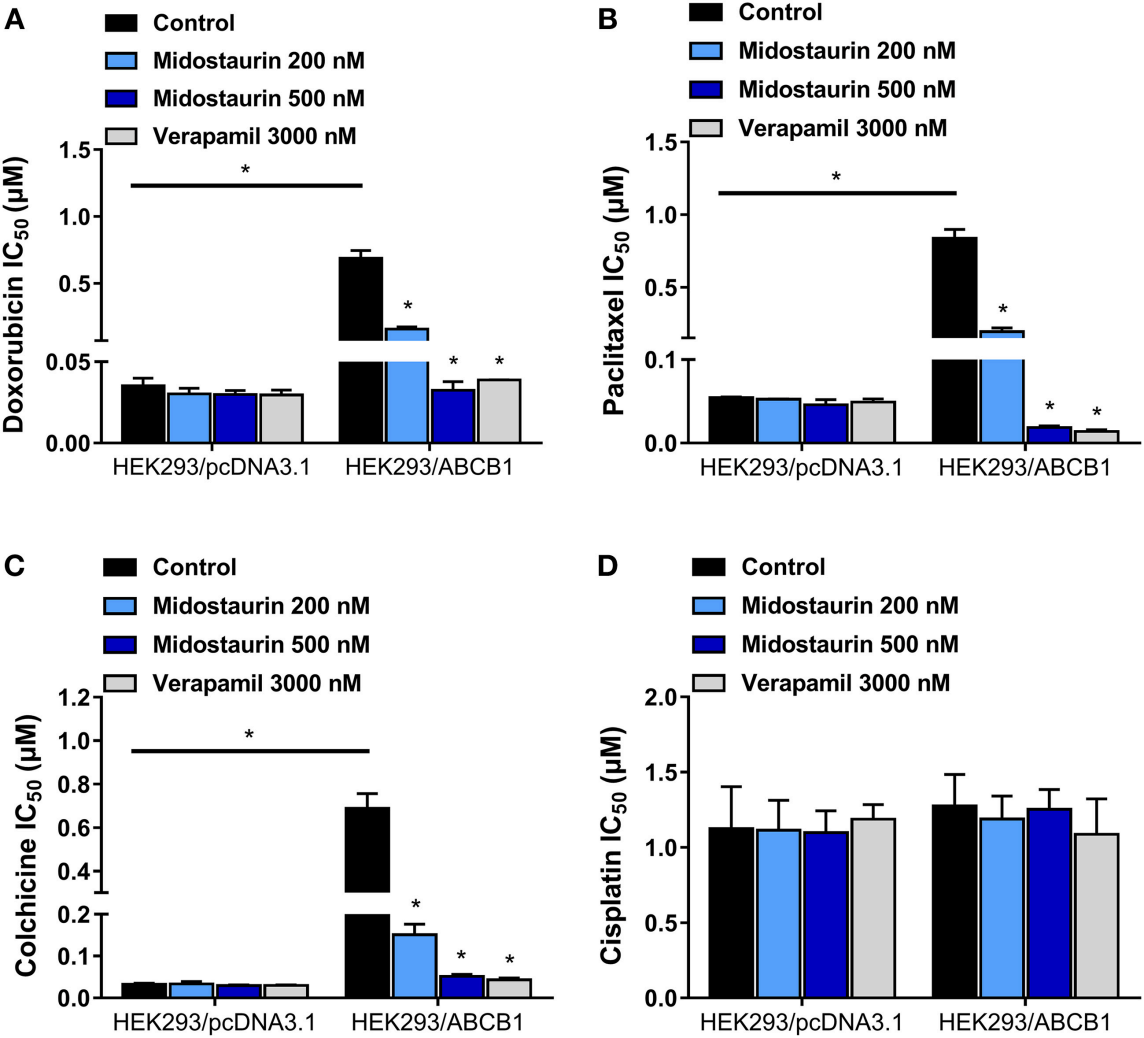
The reversal effect of midostaurin on ABCB1-mediated MDR in ABCB1-gene-transfected cells. IC_50_ values of **(A)** doxorubicin, **(B)** paclitaxel, **(C)** colchicine, and **(D)** cisplatin in parental HEK293/pcDNA3.1 and transfected ABCB1-overexpression HEK293/ABCB1 cells with or without treatment of midostaurin. Data are expressed as mean ± SD, representative of at least three independent experiments. ^*^P < 0.05, compared with control group.

We next evaluated the reversal effect of midostaurin on ABCB1-gene-transfected cells. As shown in Figure 3, midostaurin could significantly lower the IC_50_ values of ABCB1 substrate-drugs (doxorubicin, paclitaxel, and colchicine) to HEK293/ABCB1 cells at 200 and 500 nM in a concentration-dependent manner (Figures 3A–C). More importantly, midostaurin did not significantly alter the efficacy of these ABCB1-substrate chemotherapeutic drugs in parental HEK293/pcDNA3.1 cells (Figures 3A–C). Furthermore, at 200 or 500 nM, midostaurin did not significantly change the IC_50_ values of cisplatin (Figure 3D).

### Midostaurin Did Not Reverse ABCG2-Mediated MDR

**Figure 4:**
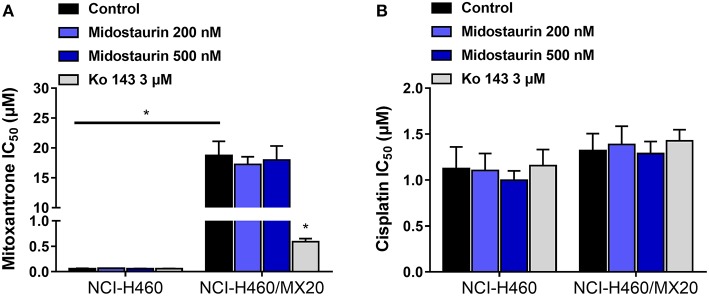
The reversal effect of midostaurin on ABCG2-mediated MDR in ABCG2-overexpression cancer cells. **(A)** IC_50_ values of mitoxantrone in parental NCI-H460 cells and resistant NCI-H460/MX20 cells. **(B)** IC_50_ values of cisplatin in parental NCI-H460 cells and resistant NCI-H460/MX20 cells. ^*^P < 0.05, compared with control group.

As shown in Figure 4, midostautrin (200 and 500 nM) could not significantly lower the IC_50_ value of mitoxantrone, a known substrate of ABCG2-mediated MDR, to drug-selected NCI-H460/MX20 cells. In this study, we chose Ko143 as a positive control drug because it is a potent ABCG2 inhibitor (21). Cisplatin was used as a negative substrate drug as previously described (25).

### Midostaurin Did Not Influence the Protein Expression Level or Subcellular Localization of ABCB1 Transporters

**Figure 5:**
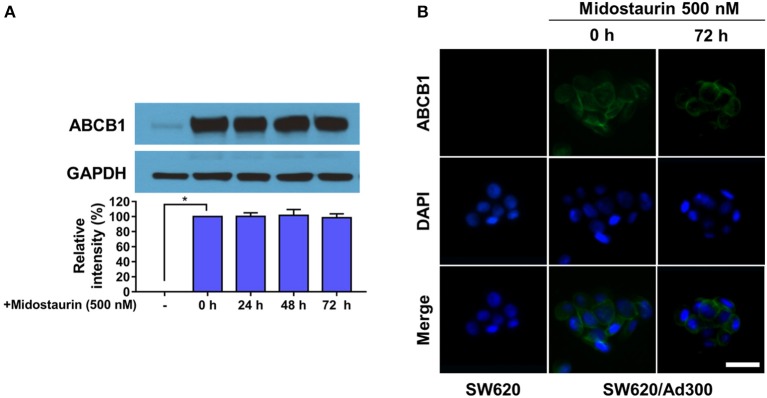
Midostaurin did not alter the protein expression and subcellular localization of ABCB1 transporter. **(A)** Detection and relative intensity of ABCB1 expression in KB-C2 cells incubated with 500 nM of midostaurin for 0, 24, 48, and 72 h. **(B)** Sub-cellular localization of ABCB1 expression in SW620/Ad300 cells incubated with 500 nM of midostaurin for 72 h. Scale bar, 100 μm. ^*^P < 0.05, compared with control group.

The next step was to figure out the mechanism of action of midostaurin. Theoretically, there are varied mechanisms involved in the reversal of ABCB1-mediated MDR. For examples, the reversal effect could be due to down-regulation of ABCB1 protein expression level and/or the change of ABCB1 transporter subcellular localization. To evaluate the effect of midostaurin on the protein level of ABCB1 transporter, we conducted Western blotting and immunofluorescence assays to detect whether midostaurin could impact the ABCB1 protein expression and/or subcellular localization. As shown in Figure 5A, after incubation for 24, 48, and 72 h, respectively, midostaurin did not significantly change the expression level of ABCB1 protein (170 kDa) in ABCB1-overexpressing KB-C2 cells. Furthermore, midostaurin did not change the localization of ABCB1 at the subcellular level after incubating for up to 72 h in ABCB1-overexpressing KB-C2 cells (Figure 5B). These results suggested that midostaurin influenced neither the expression level nor the subcellular localization of ABCB1 protein even at high concentrations.

### Midostaurin Significantly Increased the Intracellular Drug Accumulation in ABCB1-Overexpressing Cancer Cells

**Figure 6:**
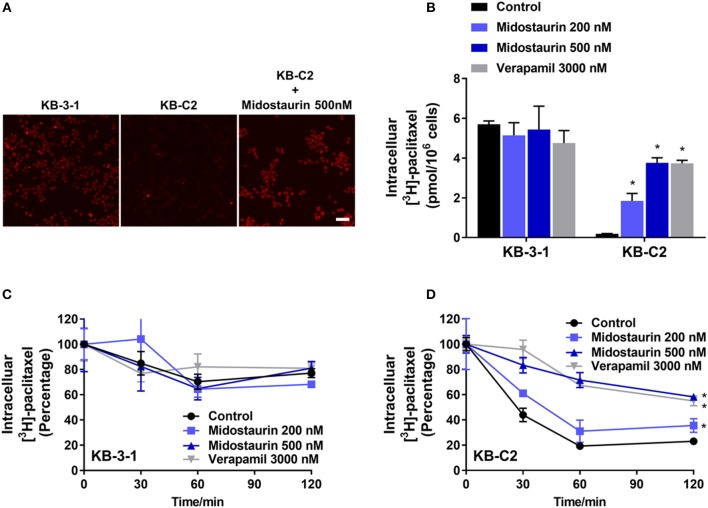
The effect of midostaurin on accumulation and efflux activity in cancer cells overexpression ABCB1 transporter. Scale bar, 50 μm. **(A)** The effect of midostaurin on accumulation of doxorubicin. **(B)** The effect of midostaurin on accumulation of [^3^H]-paclitaxel in KB-3-1 and KB-C2 cells. **(C,D)** The effects of midostaurin on efflux of [^3^H]-paclitaxel in KB-3-1 and KB-C2 cells. ^*^P < 0.05, compared with control group.

The above results indicated that midostaurin could reverse ABCB1-mediated MDR without altering the protein expression level or subcellular localization in ABCB1-overexpressing cancer cells. We then conducted our drug accumulation assay to further understand the mechanism of the reversal effect of midostaurin. Firstly, we conducted our doxorubicin accumulation assay with 500 nM of midostaurin. As shown in Figure 6A, midostaurin significantly enhanced the accumulation level of doxorubicin in ABCB1-overexpressing KB-C2 cells. We also conducted our [^3^H]-paclitaxel accumulation assay to get a digitized result. The intracellular level of [^3^H]-paclitaxel was measured in cells overexpressing ABCB1 transporter in the presence or absence of midostaurin. As shown in Figure 6B, midostaurin significantly increased the intracellular levels of [^3^H]-paclitaxel in ABCB1-overexpressing KB-C2 cells in a dose-dependent manner. However, in parental KB-3-1 cells, no significant change in [^3^H]-paclitaxel was found. In this study, verapamil was used as a positive control reversal reagent.

### Midostaurin Significantly Inhibited the Efflux Function of ABCB1 Transporter in ABCB1-Overexpressing Cancer Cells

The efflux of antineoplastic drugs through ABCB1 transporter is involved in ABCB1-mediated MDR. We conducted an efflux assay at different time points (0, 30, 60, and 120 min) to determine whether midostaurin could inhibit the efflux function of ABCB1 transporter. As shown in Figures 6C,D, midostaurin significantly decreased the efflux level of [^3^H]-paclitaxel in ABCB1-overexpressing KB-C2 cells in a dose-dependent manner, but this change was not shown in parental KB-3-1 cells. These results suggested that midostaurin could significantly increase the accumulation of anticancer drugs by inhibiting the efflux function mediated by ABCB1.

### Midostaurin Significantly Inhibited the ATPase Activity of ABCB1 Transporter

**Figure 7:**
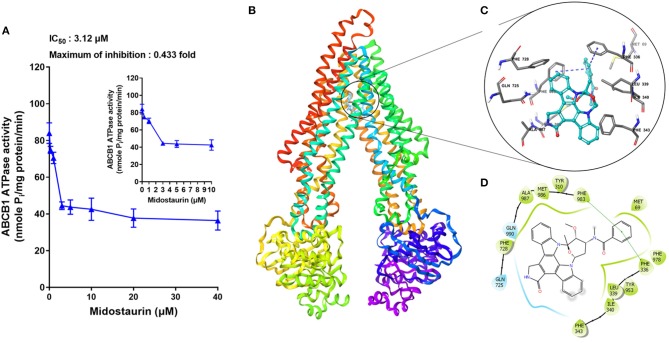
The effects of midostaurin on the ATPase activity of ABCB1 and the molecular modeling study of midostaurin with human homology ABCB1. **(A)** Effect of midostaurin on the ATPase activity of ABCB1. The inset graphs illustrate the effect of 0–10 μM midostaurin on the ATPase activity of ABCB1. Data are mean, representative of three independent experiments. **(B)** Overall view of midostainrin-ABCB1 complex. **(C)** Docked position of midostaurin within the drug-binding site of human ABCB1 homology model by Glide docking. Midostaurin is shown as ball and stick mode with the atoms colored: carbon-cyan, hydrogen-white, nitrogen-blue, oxygen-red. Important residues are shown as sticks with gray color. π-π stacking interactions are indicated with blue dotted short line. **(D)** The two-dimensional ligand-receptor interaction diagram of midostaurin and human ABCB1. The amino acids within 3 Å are shown as colored bubbles, cyan indicates polar residues, and green indicates hydrophobic residues. The green short line shows π-π stacking interaction.

The hydrolysis of ATP is the energy source of substrate-efflux mediated by ABCB1. Hence, we conducted the ABCB1-mediated ATP hydrolysis in the presence or absence of midostaurin at 0–40 μM serial concentrations. As shown in Figure 7A, midostaurin significantly inhibited the ATPase activity of ABCB1 transporters in a dose-dependent manner. The concentration of midostaurin required to obtain 50% of maximal inhibition (IC_50_) was 3.1 μM with the maximum of inhibition being 0.4-fold. These results suggested that midostaurin could inhibit the ATPase activity in ABCB1 transporters by interacting with the drug-binding pocket of these transporters. Therefore, one of the reversal mechanisms of midostraurin includes a reduced energy source for ABCB1 efflux function through inhibition of ATPase activity.

### Docking Analysis of the Binding of Midostaurin With ABCB1 Homology Model

The best-scored docked positions of midostaurin with ABCB1 transporter are shown in Figure 7. The phenol ring of the methylbenzamide moiety in midostaurin has π-π interactions with the residues Phe336 and Phe983 of human ABCB1 (Figure 7C). In addition, midostaurin has hydrophobic interactions with residues of ABCB1 including Met69, Leu339, Ile340, Phe343, Phe728, Met986, and Ala987 (Figure 7D), which stabilize midostaurin in the substrate-binding pocket of ABCB1.

## Discussion

Growing evidence has shown that the failure of clinical treatment resulting from drug resistance to chemotherapeutic drugs in a series of cancer cell lines is tightly correlated with the overexpression of ABC transporters. It has been widely reported that cancer cells overexpressing ABCB1 transporter is a key factor that could imply poor prognosis as well as low survival rate in cancer patients (26–30). Moreover, genetic polymorphisms in ABC transporters, especially in ABCB1 and ABCG2 transporters, could significantly increase the high risk of death in patients who suffer from colorectal malignancy or non-small cell lung cancer (NSCLC) (31, 32). In recent decades, many small-molecule target drugs have been reported to have the capacity to reverse ABC transporter-mediated MDR, via inhibiting the function, downregulating the protein expressing level, and/or changing the subcellular localization of ABC transporters. Previously, we have reported that selonsertib, ulixertinib, and VS-4718 can significantly antagonize ABC transporter-mediated MDR (21, 23, 33). Unfortunately, there was no successful clinical case study on the therapeutic strategies to develop ABC transporters inhibitors as reversal reagents to reverse drug resistance. Nonetheless, growing evidence has shown that the overexpression of ABC transporters is mainly involved in MDR in cancer, and it is also critical in regulating oral bioavailability of anticancer drugs and reagents. A series of first-line chemotherapeutic drugs, including doxorubicin, paclitaxel and mitoxantrone are substrates of ABC transporters, meaning that these anti-cancer drugs will be pumped out from the cancer cells and finally lead to the failure of clinical carcinoma treatment (1–4, 34). Therefore, we confirm that screening small molecules to obtain the inhibitors of ABC transporters is still a potential and effective treatment strategy to circumvent MDR in cancer.

In this *in vitro* study, we evaluated the effect of midostaurin on ABCB1-mediated MDR. We mainly found that midostaurin, at non-toxic concentrations (200 and 500 nM), can significantly overcome ABCB1-mediated MDR in a series of cancer cell lines in a concentration-dependent manner. Firstly, to avoid potential reversal effects caused by the cytostatic effect of midostaurin, we performed our MTT assays to evaluate the anti-proliferative effect of midostaurin in the cells we would use in this study. Based on the results, we conducted further reversal studies using 200 and 500 nM of midostaurin. Our reversal study indicated that midostaurin could significantly reverse ABCB1-mediated MDR in KB-C2 and SW620/Ad300 cells, which were selected by treatment with colchicine or doxorubicin, respectively. Moreover, midostaurin could not alter the efficacy of certain chemotherapy substrate-drugs in their corresponding parental KB-3-1 or SW620 cells. In addition, midostaurin could not antagonize ABCG2-mediated MDR in ABCG2-overexpressing cancer cells NCI-H460/MX20. Based on these results, we hypothesized that the reversal effect of midostaurin was specific to interactions with the ABCB1 transporter. It is notable that we then verified this hypothesis by a reversal study in gene-transfected cells. We found that midostaurin could also lower the IC_50_ values of doxorubicin, paclitaxel, and colchicine in HEK293/ABCB1 cells compared with parental cells in a dose-dependent manner, but not those in parental HEK293/pcDNA3.1 cell line. Furthermore, midostaurin did not sensitize either parental HEK293/pcDNA3.1 cell line or HEK293/ABCB1 cells to cisplatin, a drug that does not use ABCB1 as a substrate. These results suggest that midostaurin exclusively reversed ABCB1-mediated MDR.

The reversal of MDR mediated by ABC transporters may be involved in the down-regulating and/or change of subcellular localization of certain ABC transporters. However, as shown in our Western blotting and immunofluorescence assays, no significant down-regulation of ABCB1 protein was found, and all ABCB1 protein was located on the membrane of KB-C2 cells after incubating with midostaurin for up to 72 h. In other words, these results signified that the mechanisms of midostaurin on the reversal of ABCB1 mediated-MDR were not due to the down-regulating of the protein level or change of subcellular localization of ABCB1 transporters. Nevertheless, as midostaurin is a multi-kinase inhibitor, we could not fully eliminate the possibility that part of the reversal effect of midostaurin could be associated with its effect to other proteins and/or potential cross-talk with other signals, which may impact the efflux function of ABCB1 transporter, and this needs to be studied further in the future. Further study should also evaluate the potential effect of midostaurin on the protein expression level of ABCB1 with increased concentration or prolonged incubation time. Moreover, a process named post-translational modifications (PTM) plays an important role in proteins, especially transporters' function (35). It has been reported that ABCB1 could be phosphorylated at S661, S667, S671, and S683 to modulate its cell surface trafficking (36), and Pim-1 kinase could prevent ABCB1 from degradation, enabling glycosylation and cell surface expression (36). These clues indicate that it is necessary to further determine the effects of midostaurin on PTM of ABCB1 using higher concentration and/or longer incubation time.

Subsequently, drug accumulation and efflux assays were conducted, so that we could deeply understand the mechanisms of midostaurin on ABC transporter-mediated MDR attenuation. Our results indicated that midostaurin could significantly increase the intracellular concentration of ABCB1 substrate-drugs (doxorubicin and [^3^H]-paclitaxel) in ABCB1-overexpression KB-C2 cells. Midostaurin could also significantly prevent [^3^H]-paclitaxel from being pumped out of KB-C2 cells in a concentration-dependent manner. However, there is no significant change in doxorubicin or [^3^H]-paclitaxel in accumulation or efflux in parental KB-3-1 cells. These novel findings were congruent with our observed reversal effects of midostaurin. Our mechanism study also indicated that midostaurin could increase the accumulation of certain substrate-drugs (doxorubicin and [^3^H]-paclitaxel) in ABCB1-overexpression cancer cells by targeting the function of ABCB1 transporter.

ABC transporters, including ABCB1, obtain energy via ATP hydrolysis, and this can be modulated by the presence of certain substrates or inhibitors (37, 38). In our ATPase assay, we found that midostaurin could significantly inhibit the ATPase activity of ABCB1 in a dose-dependent manner, and the maximal inhibition level was 0.4-fold. Nevertheless, the accurate binding site of midostaurin with ABCB1 transporter remained unclear. In the *in silico* modeling study, we predicted that midostaurin could interact with the drug-binding pocket in the transmembrane domain (TMD) of ABCB1 transporter.

Midostaurin is an anticancer drug approved by FDA for treatment of AML with FLT3-mutant subtype (11). Over the years, the effect of midostaurin on multidrug resistance mediated by ABCB1 has been investigated independently in great detail. At the very beginning, midostaurin, also known as PKC412 and CGP41215, was developed as a PKC inhibitor, and prior work has documented the effectiveness of midostaurin in reversing MDR. Utz et al. (39) and Fabbro et al. (40) have reported that midostaurin could sensitize CCRF-VCR1000 cells and KB-8551 cells to adriamycin and vinblastine, without altering the ABCB1 mRNA expressing level. As a derivative of staurosporine, midostaurin was also identified to have the capacity to increase doxorubicin accumulation in doxorubicin-resistant cell line A2780/Adr. Meanwhile, midostaurin also acted as a reversal reagent in P-gp mediated leukemia resistance (41, 42). Budworth et al. (43) have explored the reversal effects of midostaurin on P-gp mediated breast cancer MCF7/Adr cell line resistance and Beltran et al. (44) have confirmed that such an effect of midostaurin was related to alterions in the phosphorylation of P-gp. In the study conducted by Courage et al. (45), midostaurin-resistant A549/CGP human lung cancer cell line was identified that overexpressed P-gp, indicated that P-gp may play a key role in midostaurin-mediated MDR. Another research showed that midostaurin could not affect the development of RD cells resistance (RD is a cell line that is resistant to vincristine, and has a mutant P53 but does not have detectable P-gp). This finding, combined with the above evidence, suggests that midostaurin may influence P-gp-mediated MDR (46). Moreover, midostaurin was also documented to alter P-gp efflux function and induce cell death in FLT3 ITD/P-g-positive samples (47). Ganeshaguru et al. (48) studied the effect of midostaurin on malignant cells from B-CLL (B-cell chronic lymphocytic leukemia) patients, and the results showed that nearly 1/3 of B-CLL cells that were originally resistant to chlorambucil and fludarabine were sensitive to midostaurin. P-gp-mediated efflux activity of nearly half of B-CCL cells were observed to be modulated by midostaurin. This novel finding further supported the conclusion that midostaurin could reverse ABCB1-mediated MDR. However, due to technical restrictions in protein structure identification and the incomplete functional research on ABC transporters, few studies systematically explained the exact mechanisms of midostaurin on ABCB1-mediated MDR. In this study, we conducted a series of experiments to verify the reversal effects of midostaurin on ABCB1-mediated MDR in cell lines which were not involved in other studies. Furthermore, we used human ABCB1 homology model to conduct our *in silico* docking study, through which we determined the specific residues that midostaurin would bind to, indicated the potential combining mode of midostaurin with ABCB1 transporter. On the other hand, with the development of pharmacological and molecular biology, various resistant cell lines with definite mechanisms were established and identified. It is necessary to verify the reversal effect and to determine the mechanisms of midostaurin on MDR by utilizing different resistant cell lines. More recently, the results from Hsiao et al. (49) showed that midostaurin could sensitize ABCB1-overexpression KB-V-1, NCI-ADR-RES, and NIH3T3-G185 cells to paclitaxel, colchicine, and vincristine. They also found that midostaurin could enhance colchicine-induced apoptosis effect in KB-V-1 cells, without altering the expression level of ABCB1 transporter. This finding coincides with the results we found when we co-administrated midostaurin with ABCB1-substrate chemotherapeutic drugs. In conclusion, our study demonstrates that midostaurin could overcome ABCB1-mediated MDR by directly inhibiting the efflux function of ABCB1 transporter; as a result, midostaurin can increase the accumulation of antineoplastic drugs. This novel study also suggests that co-administration of midostaurin with certain substrate-chemotherapeutic drugs of ABCB1 may benefit cancer clinical treatment by circumventing MDR. However, we should not overstate the function of midostautin on MDR before further *in vivo* study and even clinical evaluation is completed as three generations of ABCB1 inhibitors have all failed to be applicable in clinic (50). Admittedly, it remains to be determined whether midostaurin could contribute to improving chemotherapeutic outcome in clinic. More recently, a global study of the efficacy and safety of midostaurin plus chemotherapy in newly diagnosed patients with FLT3 mutation negative (FLT3-MN) acute myeloid leukemia (AML) is recruiting (NCT03512197), and a phase II clinical evaluation is recruiting for midostaurin associated with standard chemotherapy in patients with core-binding factor leukemia (AML FLT3) (NCT03686345). These clinical trials may provide more potent evidence on combined utilization of midostaurin with conventional chemotherapeutic drugs, which would make midostaurin a sensitizing drug, not just a “reversal reagent.”

## Author Contributions

DK and ZC conceived the general idea. NJ, YY, CC, JW, ZL, ZW, and QC performed experiments. NJ, ZC, DY, and DK analyzed the results. NJ, YY, CC, ZL, ZW, and QC wrote the first draft. DK and ZC revised the manuscript.

### Conflict of Interest Statement

The authors declare that the research was conducted in the absence of any commercial or financial relationships that could be construed as a potential conflict of interest.
